# Health Impacts of Wildfires

**DOI:** 10.1371/4f959951cce2c

**Published:** 2012-11-02

**Authors:** Sarah Elise Finlay, Andrew Moffat, Rob Gazzard, David Baker, Virginia Murray

**Affiliations:** Health Protection Agency; Forest Research; South East England Wildfire Group; Health Protection Agency; Health Protection Agency

## Abstract

Introduction Wildfires are common globally. Although there has been considerable work done on the health effects of wildfires in countries such as the USA where they occur frequently there has been relatively little work to investigate health effects in the United Kingdom. Climate change may increase the risk of increasing wildfire frequency, therefore there is an urgent need to further understand the health effects and public awareness of wildfires. This study was designed to review current evidence about the health effects of wildfires from the UK standpoint. Methods A comprehensive literature review of international evidence regarding wildfire related health effects was conducted in January 2012. Further information was gathered from authors’ focus groups. Results A review of the published evidence shows that human health can be severely affected by wildfires. Certain populations are particularly vulnerable. Wood smoke has high levels of particulate matter and toxins. Respiratory morbidity predominates, but cardiovascular, ophthalmic and psychiatric problems can also result. In addition severe burns resulting from direct contact with the fire require care in special units and carry a risk of multi – organ complications. The wider health implications from spreading air, water and land pollution are of concern. Access to affected areas and communication with populations living within them is crucial in mitigating risk. Conclusion This study has identified factors that may reduce public health risk from wildfires. However more research is needed to evaluate longer term health effects from wildfires. An understanding of such factors is vital to ensure preparedness within health care services for such events.

## Introduction

Wildfires, in the form of bush fires, vegetation fires, forest fires, heath and grass fires, are prevalent throughout the world. Recent high profile events in Chile 1, Australia 2 and California 3 have reminded the global community of the devastating effects uncontrolled fire may cause. The threat is closer to home too; there are on average 70 000 forest fires annually in Europe alone 4, and whilst these predominantly occur in countries with warmer climates such as Portugal and Greece, wildfires, in the form of uncontrolled burning of vegetation (albeit not full forest fires), do occur with increasing frequency in the UK 5. According to the Department for Communities and Local Government, Fire Statistics Branch, there were over 58 000 grass and heathland fires in Great Britain in 2010/11 6.

The UK Climate Change Risk Assessment (CCRA) 2012 has cited wildfires amongst the top seven risks to the natural environment in England 7. With climate change, the risk of wildfires is likely to increase and a 30% to 50% increase in wildfires by 2080 is predicted 8. The health effects of heat waves and heat exposure are well documented, but the health effects of wildfires are less widely known. Whilst much work has been done by agencies such as the Forestry Commission, the Meteorological Office and the Fire and Rescue Service to investigate how best to predict and limit damage from fires, little equivalent work has been done from the perspective of healthcare. Understanding the health impacts of wildfires and ensuring that our front line health care services are equipped to deal with them can help to reduce suffering in the aftermath of a wildfire.

**Location and size of Wildfires in England FY 2009/10 – 2010/11:**
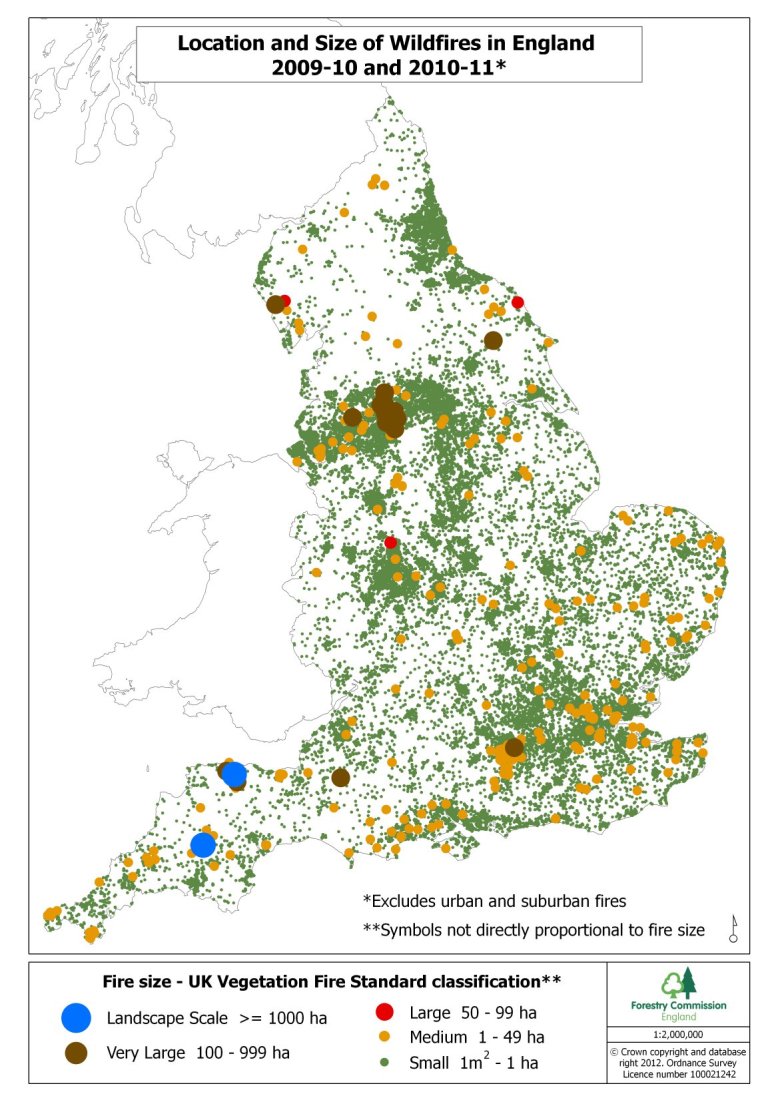
Courtesy of Forestry Commission England. Data source: Department for Communities and Local Government (National Incident Recording System)

## Aim

The object of this study was to collate and review the evidence regarding human health impacts from global wildfire experience. Understanding these impacts, and mitigation measures adopted in other countries, should help to increase awareness amongst health professionals and enable the UK to prepare effectively for the increase in wildfires that are likely to occur during this century as a result of climate change.

## Methods

**Protocol**

A search strategy was devised to study the available literature across various databases. Literature up to the end of January 2012 was included.

**Eligibility Criteria**

Inclusion criteria were:

- All papers relating to wildfires and human health

Exclusion criteria were:

- All subjective reports containing no scientific data
- Reports relating only to geographical and forestry issues around wildfires
- Literature relating to smoke and fires from sources other than vegetation

**Information Sources**

****The following databases were searched

- Medline
- Embase
- Cochrane
- Google Scholar

**Search**

**Table:** Search items included in various searches were:

Wildfires
Forest Fires
Bushfire
Grass Fire
Health
Human

These were used in varying combinations using OVID as the primary interface. The Cochrane database was searched but revealed no further relevant papers. The first 100 hits on google scholar were reviewed by title alone, which revealed some useful background literature but no further key papers.

There is no set definition of a “wildfire” but it is widely understood to mean the uncontrolled burning of vegetation. The search terms were chosen after a collective discussion between experts in the field to select the most appropriate terminology accounting for both technical and colloquial description of the above.

**Search - Examples of search using Ovid interface:** 

1	(forest and fire).mp. [mp=title, abstract, full text, caption text]
2	(Grass and fire).mp. [mp=title, abstract, full text, caption text]
3	(Wild and fire).mp. [mp=title, abstract, full text, caption text]
4	(Bush and fire).mp. [mp=title, abstract, full text, caption text]
5	health.mp. [mp=title, abstract, full text, caption text]
6	human.mp. [mp=title, abstract, full text, caption text]
7	1 OR 2 OR 3 OR 4
8	5 AND 6
9	7 AND 8
10	limit 9 to (clinical medicine and original articles)

Total hits: 424

(Hand searched by abstract to include relevant papers only)

**Study Selection**

Studies were selected by review of titles and abstracts. If full text was readily available this was also reviewed. The bibliographies of each relevant paper were hand searched for further relevant documents.

The majority of literature pertaining to this subject was in the form of epidemiological observational studies and case reports.

A total of 81 useful and relevant papers were identified from the literature searches and bibliography searches. These were mainly in English although one Spanish document was identified.

**Risk of bias in individual studies**

There is a risk of selection and observational bias within the papers chosen, but due to the paucity of original research in this area, studies were not excluded because of this risk alone.

**Risk of bias across studies**

Publication bias may also be an issue as it is likely that data from high profile wildfires is more likely to reach the public domain than that from smaller episodes. Funding bias may also be an issue as the chance of gaining sponsorship to investigate and report effects from smaller fires may be difficult.

Grey literature was reviewed and expert opinions sought. Meetings and telephone conferences were arranged with experts in the field, including representatives from the Forestry Commission and Fire Services.

## Results

**Study Selection**

A total of 76 relevant papers were identified from the literature searches and bibliography searches. These were mainly in English although one Spanish document was identified.

**Study Characteristics**

The majority of literature pertaining to this subject was in the form of epidemiological observational studies and case reports.

In light of the high incidence of wildfires, there is surprisingly little literature relating solely to their health effects. Much of the literature refers to just a few renowned events, some of which are presented below (table 1) whilst examples of recent wildfires in England illustrate the fact that although these wildfires may be smaller than those abroad, their potential impact may be significant (table 2).

**Table 1 - Case studies: reports of international wildfire events of note:** 

Location, date	Details
Sydney, 1994	Over 800 extensive bush fires spread along the coast of New South Wales in summer 1993-1994. Four people were killed - two civilians and two fire fighters, and 27 250 people were evacuated. 800 000 hectares were burnt. 225 homes and other buildings destroyed and a further 150 were damaged 9.
Indonesia, 1997	Widespread bushfires in Indonesia in 1997 (over 5 018 000 hectares 10 resulted in a haze of air pollution which resulted in severe adverse health effects in Indonesia, Malaysia and Singapore 11 .
Canada, 2003	2500 fires started in British Columbia in 2003 during a period of particularly hot, dry weather 12. Many urban areas were affected; 334 homes were destroyed and 45 000 people evacuated. The total cost of the fire storm is thought to be around $700 million (Canadian dollars). Three deaths were reported – all pilots who died when trying to put out the blaze.
California, 2007	Wildfires in Southern California in October 2007 burnt over an area of 202 300 hectares, destroying around 1 500 homes. Nine people died 13.
Victoria, Australia, 2009	Black Saturday, one of Australia’s worst natural disasters occurred on 7 February 2009, when temperatures in Melbourne reached 46.4°C (the hottest on record). Bushfires which had started earlier in the day swept across the region, blown by 100km/h winds. Over 141 600 hectares burned. 173 people died, 414 were injured 14.
Russia, 2010	In summer 2010, the western part of the Russian Federation experienced extreme heat and severe wildfires. More than 20 000 forest fires over an area of 2800 km2 were recorded, emitting high levels of carbon monoxide and particulate matter. Cumulative excess deaths in July and August of 2010 amounted to 54 000 compared to the same period in 2009 15.

**Table 2 - Case studies 2: Examples of recent wildfires in England:** 

Location, date	Details
Swinley Forest, May 2011	May 2011 saw one of the biggest English wildfires – in Swinley, Berkshire 16. Over 105 hectares of forest burnt, fanned by strong winds on a background temperature of 30°C 17. Several local schools were closed due to the risk of smoke induced illnesses. The fire spread close to Broadmoor high security hospital, and urgent plans for evacuation of patients were made. Several local homes adjacent to the site were evacuated.
Pitbright Ranges, 2003 and 2010	In 2003 and 2010 over 850 hectares of lowland heath burnt at Pirbright Ranges 18, owned by the Ministry of Defence. Again high temperature, low humidity and strong winds created a large fire front, at times 2 km wide. Along with road closures and significant local disruption, military housing adjacent to the ranges was evacuated and smoke threatened a large housing estate to the west with vegetation ash landing several km away in Farnborough and Aldershot.
Thursley Common, 2006	Natural England’s National Nature Reserve in Surrey burnt in 2006, the wildfire covering over 160 hectares 19. As well as threatening several private properties adjacent to the site and closing several roads, three fire fighters suffered burns requiring hospital treatment.

Results from the searches can be summarised as follows:

- *Health threats and the Source-Pathway-Receptor model.* This is a model of the means by which wildfires and health concerns can be explored further (see below).
- *Toxicology of wildfire smoke.* Some research has been done looking at the toxicology of wildfire smoke. Identifying its toxic components could help improve our understanding of adverse health effects caused. Particulate matter from wildfire has been shown to differ from other sources of particulate matter, of relevance to our understanding of its toxicology.
- *Health effects. *Many systems are affected by wildfire smoke, predominantly through the respiratory system. Cardiovascular effects and ocular problems can also occur as well as acute burns. Psychological and psychiatric effects can be significant in relation to larger fires.
- *Water and land pollution. *Both water and soil pollution can cause longer term threats to human and ecosystem health after a wildfire.
- *Resource and access. *The effect that a wildfire has on access to healthcare services and vital resources can adversely affect health. Serious wildfires could overwhelm local healthcare resources unless a clear plan is in place.
- *Communication. *The importance of effective communication in mitigating against adverse health effects is emphasised.

## Health Threats: source-pathway-receptor model

The Source-Pathway-Receptor Exposure Model, used for risk assessing the impact of acute and chronic chemical exposures on health e.g. in flooding and contaminated land studies 20 can also be used to examine the potential health effects of wildfires.

**Source**

Heat and smoke from burning vegetation can cause adverse health effects. The majority of UK wildfires are small and contained, but as shown in 2010 and 2011 larger, more landscape scale incident can occur. The most significant land use type of areas burnt were open habitats (mountain, moor, heath and grasslands). Types and amount of fuel, past and present weather conditions and topography can all affect fire intensity, and thus the degree and type of health effect 21. Even within England, sources of fuel for wildfires vary significantly (Figure 2).

**Land types burnt in wildfires FY2009/10-2010/11:**
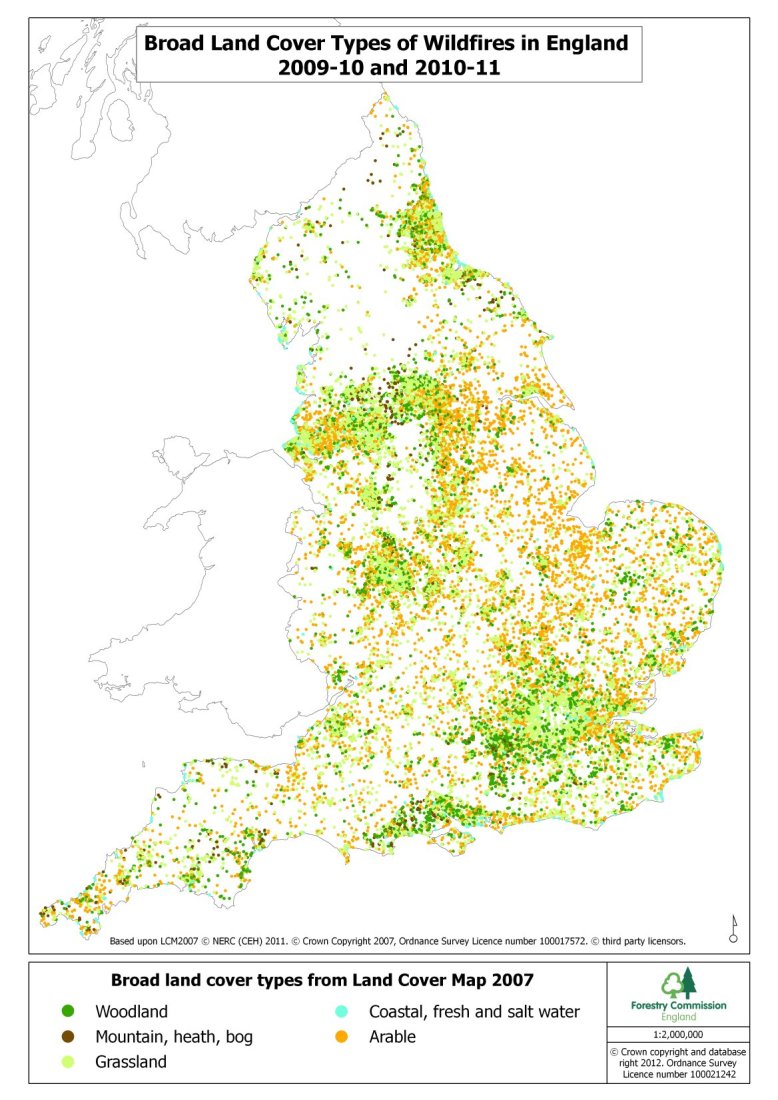
Courtesy of Forestry Commission England. Data source: Department for Communities and Local Government (National Incident Recording System) 22

**Pathway**

Four main exposure pathways exist:

- Direct exposure to the flames and radiant heat;
- Exposure to smoke from burning or smouldering material dispersed through the air;
- Exposure to Land/soil contaminated by the chemical products of burning vegetation, from soil erosion caused by vegetation removal during the fire, or from suspended dust dispersed through the air;
- Water contamination, caused by particulate matter deposition on water or leachates from the land directly affected by fire.

**Receptor**

The receptors in this study are humans who live in the vicinity of the fires who may experience adverse health effects. The vulnerability of the population to potential harm depends on many factors 23, most notably:

- Prevalence of demographically vulnerable groups such as those at the extremes of age, pregnant women, those of poor socio-economic status
- Prevalence of pre-existing disease in the community (especially cardiac and respiratory)
- Access to publically - available information on risk mitigation
- Emergency Preparedness including the presence of an early warning system
- Location of dwellings, workplaces and critical infrastructure in relation to the fire, and access/egress from them.
  - An example of this is the 2011 Swinley Wildfire in Berkshire that threatened buildings including Broadmoor Psychiatric Hospital (Case studies 2).

Vulnerable populations are more likely to suffer adverse effects from wildfire smoke, thus actions should be taken to mitigate risk where possible, for example by ensuring clear access to reliable public information and a robust system of emergency preparedness.

**Toxicology of wildfire smoke**

Wildfire smoke consists of particulate matter and gaseous products of combustion. Adverse health effects, including an increase in daily mortality, have been linked to air pollution associated with bushfires and dust storms. A recent Australian study looking at the effects of bushfires between 1997 and 2004 illustrates this 24: A 5% increase in non-accidental mortality (OR 1.05 (95%CI: 1.00–1.10)) was observed on days of high air pollution from bushfire smoke.

Particulate matter is the predominant air pollutant seen in bushfire smoke, caused especially by the burning of vegetation and wood 25. PM_10 _particles (which are able to pass through the upper respiratory tract and are deposited in the airways), and PM_2.5_ particles (may be respired deeper within the lungs and deposited in the gaseous exchange region of terminal bronchi and alveoli) are produced by burning vegetation. Health effects of particulate matter are well documented: a 0.5-2% increase in mortality with each increase of 10 μg/m^3 ^ of urban PM_2.5 _has been observed 26. COMEAP have reported a 6% (2- 11 95%CI) increase in all cause mortality associated with this increase in PM_2.5 _.

Gaseous emissions including carbon monoxide, nitrous oxides and benzene are produced 27, as are carcinogens including polycyclic aromatic hydrocarbons (also be present on particulate matter), aldehydes, and volatile organic compounds 28. These compounds would also be expected to be adsorbed on the surface of the particulate products. Levels of ozone and nitrous dioxide are also seen to rise 27. Wildfires often have a high proportion of smouldering fuel (i.e. thermal breakdown of fuel in a normal oxygen level without flame). This is a form of incomplete combustion and is likely to produce high levels of toxins 29. As the fires are open air, the direct health effect of these toxins is likely to be low as their concentration is quickly dispersed, and toxins such as carbon monoxide are unlikely to cause immediate clinical concern. However, the longer term health effects from low level exposure to aldehydes and other carcinogens from bushfire smoke may remain a cause for medical concern.

Tan et al. 30 monitored white blood count levels in humans after the Indonesian wildfires (case studies 1) and found increased levels of polymorphonucleocytes indicating possible increased bone marrow activity which may be linked (although inconclusively) to the toxins in wildfire smoke.

**Studies of the effects of particulate matter in wildfire smoke**

PM_10_from wildfires appear to have different effects on health than urban PM_10_. An 8 year study investigating air pollution levels, including those from bushfires, and hospital admissions showed that a 10 µg/m^3^ increase in bushfire (but not urban) PM_10_ was associated with a 1.24% increase in all respiratory admissions, a 3.80% increase in COPD admission and a 5.02% increase in adult asthma admissions. Increased levels of urban PM_10_ were associated with an increase in all-cause and cardiovascular mortality but not respiratory mortality 31.

Studies from Darwin, Australia, are particularly useful as there is very little background urban PM_10_ pollution and therefore most rises in air pollution are secondary to bushfires 32. Studies from this area 33 ^,^ 34 report a significant increase in asthma and COPD presentations associated with raised PM_10_ levels from bushfire smoke.

The difference between urban and wildfire smoke is also illustrated in a study looking at the effect on macrophages exposed to wildfire and urban smoke in a murine model 35. This showed that although cytokine production in response to wildfire smoke was lower than with urban derived particles, there was increased inflammatory (determined by measuring proinflammatory cytokines) and cytotoxic activity (as measured by biochemical markers of toxicity, apoptotic activity and nitrous oxide production) per cubic metre of air containing wildfire particles than with air containing only urban particulate matter. This was probably as a result of a higher concentration of PM_10_ particles in the wildfire smoke (10.3µg/m3 compared with 5.5µg/m3 in urban air). This increased particulate size means that particles can accumulate in the lung more easily, which may have public health implications.

Wood smoke particles have also been shown to cause an inflammatory response in otherwise healthy humans. Ghio et al.’s 36 study of 10 human volunteers exposed to woodfire smoke showed an increased level of blood neutrophils, and a neutrophilic influx into the lung from bronchial and bronchoalveolar lavage samples. Although it was a small study, the authors suggest that systemic and pulmonary inflammation in human subjects can result from exposure to wood smoke particles.

Firefighters are at particular risk of inhalation of wildfire smoke particles. In recognition of this, suitable filters need to be used in breathing apparatus., such as the POVF (particulate/organic vapour/formaldehyde) filter 37.

A crude extrapolated estimate from Finland suggested that high PM_2.5_ levels following wildfires in 2002 caused additional total mortality of 9-34 cases in a population of 3.4 million compared with what would normally be expected 38. High levels of PM_10_ (both urban and from bushfires) were associated with a 1.8% increase in ED attendances in a study in Victoria (carried out during a bushfire season in 2003-2003) 39. A system of monitoring air pollution during and after wildfire events may provide useful public health information, facilitating preparedness for increased pressure on health care services, and should be considered. It would be important to monitor PM_2.5_ levels, not just PM_10_ levels. PM_10_ monitoring alone may not adequately represent the adverse effects of air quality that may be caused by the PM_2.5 _fraction 38.

## Other Health Effects

Health effects of wildfires are wide ranging, and may result directly from both the thermal effects and smoke. Further health implications include: psychological reactions to an extreme event, physical concerns such as trauma during evacuation, and pressures on local resources, from increased demand on health services and inability of patients with chronic health care conditions to access healthcare facilities.

**Respiratory symptoms**

Certain population groups are at particular risk of respiratory effects from bushfire smoke, including young children, those with pre-existing cardiopulmonary conditions, and smokers 40.

Patients with COPD have been noted to be at increased risk as a result of air pollution. A study looking at symptoms of 21 local patients with COPD in the two months following the Denver wildfires of 2002 revealed that dyspnoea, cough, chest tightness, wheeze and sputum production all increased on days when PM_2.5_, PM_10_ and carbon monoxide levels in the atmosphere increased, thus illustrating the link between air pollution from wildfires and COPD exacerbation 41.

In a paediatric cohort study 32 children with a history of wheeze suffered adverse effects from increased PM_10_ particles after the 2009 Australian bushfires, although whilst symptoms of evening wet cough worsened, dry cough and wheeze did not 42.

A study of 465 non-asthmatic teenagers affected by 2003 wildfires in Spain revealed that healthy patients with estimated smaller airways who performed on the lowest quartile of lung function tests were more susceptible to the respiratory effects of wildfire smoke 43. Those with smaller airways and poorer pre- existing lung function were more vulnerable to smoke effects.

Data from the 1994 Sydney Bushfires show that there was no increase in acute asthma related admissions in central Sydney 44 or Western Sydney 45 in the aftermath of the fires. This may not however reflect the true prevalence of asthma exacerbations, as only the more severe cases would present to the ED. In the days following the 1987 Californian bushfire, there was a 40% increase in ED attendances 46.

A cohort study from Darwin Australia shows that studies looking at hospital attendances alone may underestimate the respiratory symptoms^47^. In this study, 251 adults and children were asked to keep a record of their asthma symptoms during a 7 month bushfire period in 2004. During this time, PM_10_ ranged from 2.6 – 43.3 µg m^−3^. High PM_10_ levels were significantly associated with an onset of asthma symptoms, use of oral steroid medication, the mean daily symptom count and the mean daily dose of beta 2 agonists. However, there was no increase in the numbers of health care attendances or severe asthma attacks

An increase in respiratory symptoms and deteriorating lung function was also seen in a study of findings reported by respiratory physicians and governmental reports in Indonesia at the time of the Indonesian bushfires of 1997 48. Worsening of respiratory symptoms were seen during the same period in surrounding countries such as Malaysia 49 and Singapore 50, illustrating the ability of particulate matter and air pollution to spread widely. 94% of the air particles noted in Singapore in the haze following the Indonesian fires were PM_2.5_, and emergency department attendances related to the haze increased although overall hospital admissions due to respiratory effects did not^50^. These effects were observed more than 500 km from the bushfires.

Delayed health effects may also occur: one study looking at health effects after the 2003 Canadian wildfires showed that there was no increase in presentations to medical services for mental health or cardiovascular problems, but there was a peak in respiratory consultations 5 weeks after the fires. This may be because of delayed respiratory health effects of wildfires smoke 51. In addition, long term exposure to particulate matter may increase susceptibility to infection possibly through an impairment of respiratory clearance systems 52, thus helping to explain an increase in pneumonia and acute bronchiolitis seen after the 2003 Californian wildfires.

Despite frequent fires in Europe, very little literature exists on potential associated health effects. The vegetation fires which surrounded Vilnius in Lithuania in 2002 caused increases in hospital attendances for respiratory conditions and asthma 53. These peaked in September (the fires started in early August), possibly due to delayed respiratory effects or increased air pollutant levels. High levels of ozone, PM_10_, nitrogen dioxide and carbon monoxide were noted in the atmosphere. Possible recommendations may be to administer steroids more readily than usual for asthmatics, and recommend the avoidance of outdoor activities for those vulnerable to respiratory pathology 52.

Although these studies on the respiratory effects reported from wildfires have been undertaken, little data on the levels of pollutant exposure has been documented reducing the ability to extrapolate this information to UK wildfires.

**Burns**

An obvious risk to those in very close proximity to wildfires (most likely those failing to vacate the fire area or fire fighters) is that of direct flame burns and thermal burns. Clinical management will be similar to that of a normal burns patient where there has also bee inhalation of combustion products. Patients admitted to the ICU with burns after the Victoria Wildfires differed from “usual” burns patients in that the degree of early multi-organ failures and the severity of inhalational burn were higher than expected for the degree and percentage of burns 14. Another major factor is the number of casualties and potential demand on healthcare resources, depending on the scale of the fire and its location in relation to urbanisation.

Severe burns require specialist multidisciplinary resources, which could become overwhelmed in the case of severe wildfire. National burns disaster plans, such as the Australian Mass Casualty Burn Disaster Plan, instigated at the time of the “Black Saturday” disaster in Victoria, Australia 54, can help to mitigate against problems of overwhelmed resources.

In the 72 hours following the 2009 Victoria Wildfires, 17 patients presented to local EDs with burns of >10% of body surface area (BSA) and another 129 with burns of <10% of BSA 14. 20 patients were managed at the specialist tertiary adult burns centre, 19 of whom needed surgical procedures (such as escharotomies or debridements). The total theatre time for these burns patients in the first 72 hours was 48.7 hours 14. This illustrates the extra impact on resources of a surge of burns patients; even a small number of casualties can require significant resources.

If there are large numbers of burns presenting to hospital, careful triage is mandatory. Initial measures such as fluid resuscitation, analgesia and covering affected areas with cling film can be instigated in the ED relatively easily even to larger numbers of casualties. Such triage will avoid overloading specialist burns centres with the more minor burns that can be effectively treated locally. The triage category for the more severely injured may also have to be adapted; for example, under the Australian mass casualty burns disaster plan, the threshold of burns for admittance to the specialist burns centre (>20%BSA) was revised to BSA >30% in order to avoid overwhelming specialist services 14.

**Heat Induced Illness**

Heat induced illness can be caused by working in hot and humid conditions and will be affected by proximity to the fire. The extent of heat related illness will not be covered in full in this paper except to note that those directly involved with fire fighting are particularly vulnerable.

Modern fire fighters’ personal protective equipment (PPE) is comprised of tunics and leggings. The retention of heat within the structural PPE can cause heat related illness, initially heat exhaustion leading to heat stroke. Careful design of wildfire PPE, rest periods, adequate hydration and health awareness of those involved is extremely important 55.

**Cardiovascular effects**

There was a slight increase in admissions to the ED with ischemic heart disease on days when there was air pollution from Sydney wildfires 31. Cardiovascular mortality rates also increased on days with high levels of bushfire smoke in a study of Sydney air pollution between 1997 and 2004 (OR 1.10 (95%CI: 1.00–1.20))^56^. This may be secondary to high levels of PM_2.5 _but further research needs to be carried out to establish this link.

A similar picture was seen after the 2003 Californian wildfires 52, when a 6.1% increased rate admission for cardiovascular complaints was seen following the fires, including an 11.3% increased rate of admission due to cardiac failure in comparison to the air pollution levels reported before the wildfires started.

After the Darwin wildfires, an increase in cardiovascular complications was seen in the Australian indigenous population only, with a 3 day lag after raised bushfire smoke levels 34.

**Ophthalmic effects**

Eye irritation from air pollution has been noted, as well as reduced visibility as a result of ambient smoke 57. Reduced visibility from wildfire smoke has caused fatal road traffic collisions, so care must be taken when travelling around areas near wildfires 58. The police and highway agencies have an important role in restricting access to at risk areas.

Corneal abrasions have also been reported; 13% of patients fire-related presentations to the emergency department the week following Almeda County wildfires in California 1991 had corneal abrasions 59.

**Psychological effects**

Overseas studies demonstrate that large wildfires can be devastating, destroying not only lives but livelihoods, homes and communities. This is strikingly illustrated by Mcfarlane et al.^60^, who looked at the mental health impact of bushfires in an Australian community. Twelve months after the fires, 42% of the population exposed to wildfires were classified as potential psychiatric cases (scored according to the General Health Questionnaire 61) – more than double that seen in the non-exposed population.

A study of 357 patients who sought healthcare assistance (therefore not a random sample of exposed persons) after the 2003 Californian wildfires also gives a dramatic picture, with 33% showing symptoms of major depression and 24% showing symptoms of Post Traumatic Stress Disorder 62. Property damage and physical injury during the fires were significantly – associated with psychopathology. This suggests that screening people who present for emergency relief centres in the aftermath of a wildfire may be of help to identify people who are particularly likely to suffer from psychopathology.

A cross sectional case control study 63 looked at those affected by the Greek wildfires of 2007. Increased symptoms of somatisation, depression, anxiety, hostility, and paranoia were found in those who were victims of the fire compared with controls. Another study of 30 adults 6 weeks post exposure to wildfires showed increased levels of posttraumatic stress, depression, and anxiety symptoms 64.

There is strong psychological link between local populations and their geographical surroundings, which assume great cultural, social and personal significance. The devastating effects of a wildfire on physical surroundings can translate to psychological distress 65. Immense efforts to preserve buildings of importance to the community were made by Greek firefighters in the August 2007 fires who saved the temples of Ancient Olympia (home of the Olympic Games). A study of the mental health of these firefighters noted that 19 of 102 had post-traumatic stress disorder as defined by ICD-10 66. The authors note that early detection of post traumatic stress may help to mitigate against post disaster psychiatric morbidity.

Increased smoking and anxiolytic use has been observed after wildfire exposure. 2063 adolescents and young adults were assessed to see whether exposure to a traumatic event, in this case the 2003 Australian wildfires, increased tobacco smoking 67. Exposure to traumatic events during the disaster, independent of PTSD symptoms, was a predictor of increased tobacco use (OR 1.12, 95%CI 1.03-1.21). Increased consumption of anxiolytics was noted in men after exposure to the 2006 wildfires in Northern Spain 68.

The nature of the media coverage following a wildfire can make a difference to the population’s psychological health. Vicarious traumatisation – i.e. symptoms suggestive of post-traumatic stress disorder in patients who themselves have not been exposed to the tragedy directly but only through exposure to the media has also been noted following the 2001 New South Wales bushfires 69.

Studies of rescue workers show that psychological effects can be both delayed and in some cases beneficial. 469 firefighters were followed up for 25 months after exposure to a bushfire 61 ^,^ 70. Delayed and chronic psychiatric morbidity was more prevalent than acute morbidity, and severity of morbidity was linked to the firefighters’ losses and extent of exposure. Those involved in the recovery may show some beneficial as well as detrimental psychological effects, since team building and working together have been described 71.

Paediatric Psychological Morbidity

Many studies have focused primarily on paediatric psychiatric morbidity in relation to wildfires. Younger children are at especially high risk of PTSD symptoms, as are children who perceive their own lives to be at risk or experience ongoing loss or disruption 72. This was identified in a follow up study of 155 8-18 year olds exposed to the Canberra wildfires in 2005, leading the authors to advise that identifying and supporting younger patients and those who are experiencing ongoing disruption may help to mitigate against their development of PTSD symptoms.

Risk factors for depressive illness were assessed in a study of 2379 school children exposed to wildfires in Australia who were followed up for 6 months after the event 73. Factors contributing to psychiatric morbidity (as measured by increased emotional stress and anxiety) included evacuation and experience. These factors were also predictive for ongoing emotional distress, as was a perceived threat to self or parents.

Continuation of psychological morbidity in to adulthood may be of concern, as illustrated by a cohort of 806 children who had been exposed to bushfires in Australia in 1983 who were followed up for 20 years 74. Results suggested that although the impact of the bushfires on overall adult psychiatric morbidity was small, after 20 years, seventy-five per cent of the bushfire-exposed group reported some degree of distress in relation to the bushfire exposure.

A longitudinal study of children after Australian bushfires indicated that the mother’s psychological reaction to the disaster had a greater impact on the child’s psychiatric morbidity than the child’s own exposure to the disaster. Thus targeting mothers when offering psychological support may be worthwhile 75.

## Water and Land Pollution

Examination of potential water contamination in areas surrounding the Lithuanian fires of summer 2002 show that in the autumn of that year there was a substantial (60-81%) increase in heavy metal (copper, lead and zinc) levels in surrounding rivers 76.

Ash debris following the Californian wildfires of 2007 was found to contain high levels of heavy metals, including arsenic, cadmium, copper, and lead. A national clean up campaign was organised because of concerns that exposure to high levels of such metals could cause long term health effects 77.

After the Russian wildfires in 2010, concern was raised that up to 4% re - suspension of radioactively contaminated (from Chernobyl) soil could occur in areas affected by wildfire. Increased levels of caesium, strontium and plutonium occurred 78. However the associated health risk to the firefighters and the general public was thought to be negligible.

## Resources and Access

Access and egress routes to local hospitals may be blocked by traffic congestion as people leave the wildfire area or by the fire itself. Two hospitals were in the direct line of the 2003 San Diego Wildfire, and had to prepare for complete evacuation of the hospital at very short notice 79. Healthcare workers in these hospitals suffered adverse health consequences from ash and smoke in the hospital ventilation system. Some were forced to decide between responding to the hospitals’ calls for more assistance and the need to evacuate their own homes.

Difficulty in accessing commodities including food and regular medication can have a significant impact, particularly on patients with chronic health conditions who may be unable to collect their normal medication or attend medical appointments. The elderly, the isolated, and those with chronic health problems will be particularly vulnerable. The US document “Wildfire Smoke, a guide for public health officials” advises that patients have at least a 5 day stock of medication available, as well as several day’s worth of non-perishable food 80.

As homes can be caught up in wildfires, it is imperative that access routes for inhabitants are as safe as possible, and well signposted, especially in rural areas, so that emergency services can find them easily if needed. A leaflet published by the Scottish Wildfire forum highlights this point, advising clear signage to rural properties 81. Power supplies may be disrupted, compounding effects on the local population. There is also a risk of electrocution from fallen power lines arcing because of water and smoke.

## Communication

A Global Early Warning System for Wildland Fire has been proposed 82. Spanish systems of satellite surveillance have been trialled with success, and may be worth developing elsewhere 83. Safety measures to the public faced with a wildfire threat can be issued.

An example of such a public health message is the “Ready, Set, Go” campaign in Texas 84:

- Be ready for a fire threat
- Have situational awareness if a fire threat occurs and be “set” to leave if you need to
- Go early - leave at risk areas early

The UK government’s advice 85 to “Go in, stay in, tune in”, although aimed at general emergencies, may be useful to prevent exposure to air pollution from fire smoke, as sheltering can reduce exposure 86. Obviously this is less advisable for those who are in the direct path of the fire, who may need to evacuate.

## Development Planning

In the UK the new National Planning Policy Framework 87 provides scope to improve wildfire resilience in new and existing developments under both natural hazard and climate change (mitigation and adaptation) policies. This will include:

- Residential, commercial and industrial properties,
- Nursing / care homes,
- Health care facilities (hospitals, care centres),
- Schools and other educational facilities,
- Emergency service centres,
- Transport infrastructure (road, rail, air and inland waterways etc.)
- Utility infrastructure (generation and movement of; water and sewage, gas, electricity, fuel, communications etc.)
- Other National and critical infrastructure facilities, structures and properties identified on National and Community Risk Registers

Developments, facilities, structures and properties that adjoin high risk habitats, land uses and/or landscapes are within the ‘Urban / Rural Interface’. Where wildfire could be a risk to human life it must be mitigated within the Local Authority’s Local Development Framework and agreed by the appropriate agencies and authorities.

## Summary of pointers to good practice

This study identifies the main health protection issues to be considered in the event of wildfire. It points to evidence based actions in response to acute events and ways to prepare for a potential increase in wildfires uedue to climate change. The issues highlighted below can be used as guidance in formulating plans to mitigate against risk to health from wildfires.

Respiratory health impacts

Emergency services and GPs should be prepared for increasing numbers of patients attending with **respiratory symptoms.**

- Those with chronic respiratory illness may experience a worsening in their respiratory symptoms.
- There may be an increased incidence of mild respiratory symptoms amongst previously healthy individuals, which may require some medical treatment.
- Increased doses of anti-inflammatory and bronchodilator medication may be required. Stocks of drugs should be sufficient to accommodate for this.

Minimising exposure to smoke

Considering the potential toxicity of wood fire smoke, it is advisable to **minimise exposure:**

- Air quality reports should be checked. These may have the potential to be used in conjunction with syndromic surveillance to understand health effects and their link with air pollution.
- Indoor air should be kept as unpolluted as possible by keeping windows and doors closed and shutting off external ventilation.

Other Systemic Health Effects

- **Burns **may pose a significant problem.
  - In severe fires systems should be in place to cope with increased pressure on resources needed to manage burns patients
  - Careful triage and judicious use of specialist burns services is needed
- **Cardiovascular morbidity **may increase – Emergency Departments and GPs should be aware of this.
- **Psychological effects **may be significant.
  - Support should be available to vulnerable groups
  - Responsible media reporting of events is important

Access and Egress

**Access **to homes, health care facilities and resources may be impeded.

- Systems should be in place to ensure delivery of medication and provisions to those who need them, especially vulnerable groups.
  - People living in areas prone to wildfires may be advised to keep a stock of 5 day’s worth of non perishable provisions and medications.
- Measures to maximise safety of routes to and from vulnerable areas should be in place.
- Housing and evacuation routes in rural areas should be clearly signposted.

Water and Land Pollution

Water and land near the fire site may become polluted by substances present in wildfire ash.

- An assessment of land and water pollution with remediation may be necessary.
- Local authorities will be responsible for cleaning – up operations in conjunction with the Environment Agency

Visibility

**Visibility** can be problematic

- Road users should be made aware of the potential for low visibility when driving
- Anyone presenting with eye irritation should be screened for corneal abrasion

Communication

**Good communication** is vital

- Public health information should be clear and as accurate as possible
- An **early warning system **should be in place to allow communities to prepare for wildfires and, if necessary, evacuate threatened areas
  - This may be enhanced using satellite data as has been used in Spain
  - Early surveillance and models for fire prediction would also be useful
- People with pre-existing health conditions should be made aware of the potential adverse health impact of wildfire smoke. For example asthma sufferers could be advised to increase their medication if they are likely to be exposed to smoke.

## Areas for Further Work

Relatively little work has been published regarding health effects of wildfires, in the UK despite their frequency although more has been published for events abroad.

Studies focusing particularly on air pollution from wildfires within the UK could be of use, as pollution from UK fires may vary from that found elsewhere in the world.

Health care workers treating casualties from wildfires should be encouraged to publish case studies of health effects to increase the evidence base available to the international medical community dealing with wildfires. Long term longitudinal studies looking at health effects in populations exposed to wildfires are needed as this will help to determine the level of pollution causing adverse health effects.

## Conclusion

Wildfires can cause significant health effects both in the population in the immediate vicinity and in those further from the fire (predominantly from the effects of air pollution). Simple public health advice can help to mitigate risk to health. With an increasing risk of wildfire in the UK, health care workers such as general practitioners, respiratory and emergency physicians need to understand more about the health risks of wildfires.

More research is needed to evaluate long term health effects from exposure to wildfires, and careful identification and follow up of those exposed could help in this process. The better our preparedness for wildfires in the UK, the more we can do to mitigate against their adverse health effects.

## Competing Interests

The authors have declared that no competing interests exist.

## Appendix A

### Glossary

**Table:**

BSA	Body Surface Area
CCRA	UK Climate Change Risk Assessment 2012
CO	Carbon Monoxide
ED	Emergency Department
ICU	Intensive Care Unit
PM 10	Particulate Matter with a diameter of 10 micrometer or less
PM 2.5	Particulate Matter with a diameter of 2.5 micrometer or less
WBC	White Blood Cell

## Appendix B

### Prisma Checklist

Download PDF
